# pH‐EVD: A pH‐Paper‐Based Extraction and Visual Detection System for Instrument‐Free SARS‐CoV‐2 Diagnostics

**DOI:** 10.1002/anbr.202100101

**Published:** 2021-12-07

**Authors:** Xiong Ding, Ziyue Li, Lori Avery, Enrique Ballesteros, Rohit Makol, Changchun Liu

**Affiliations:** ^1^ Department of Biomedical Engineering University of Connecticut Health Center 263 Farmington Ave Farmington CT 06030 USA; ^2^ Department of Pathology and Laboratory Medicine University of Connecticut Health Center Farmington CT 06030 USA

**Keywords:** instrument-free onsite diagnostics, nonbleeding pH paper, RNA extraction, SARS-CoV-2, visual isothermal amplification detection

## Abstract

The ongoing pandemic of coronavirus disease 2019 (COVID‐19) caused by severe acute respiratory syndrome coronavirus 2 (SARS‐CoV‐2) has caused millions of deaths worldwide. However, most SARS‐CoV‐2 detection methods depend on time‐consuming sample preparation and large detection instruments. Herein, a method employing nonbleeding pH paper to achieve both RNA extraction and visual isothermal amplification is proposed, enabling rapid, instrument‐free SARS‐CoV‐2 detection. By taking advantage of capillary forces, pH‐paper‐based RNA extraction can be accomplished within 1 min without need for any equipment. Further, the pH paper can mediate dye‐free visual isothermal amplification detection. In less than a 46‐min sample‐to‐answer time, pH‐paper‐based extraction and visual detection (termed pH‐EVD) can consistently detect 1200 genome equivalents per microliter of SARS‐CoV‐2 in saliva, which is comparable to TaqMan probe‐based quantitative reverse transcription PCR (RT‐qPCR). Through coupling with a chemically heated incubator called a smart cup, the instrument‐free, pH‐EVD‐based SARS‐CoV‐2 detection method on 30 nasopharyngeal swab samples and 33 contrived saliva samples is clinically validated. Thus, the pH‐EVD method provides simple, rapid, reliable, low‐cost, and instrument‐free SARS‐CoV‐2 detection and has the potential to streamline onsite COVID‐19 diagnostics.

## Graphical abstract

A pH‐paper‐based extraction and visual detection (termed pH‐EVD) method is developed for instrument‐free severe acute respiratory syndrome coronavirus 2 (SARS‐CoV‐2) detection. Nonbleeding pH paper is used for both RNA extraction and visual isothermal amplification detection. Through coupling with a chemically powered smart cup, the pH‐EVD method provides simple, rapid, reliable, low‐cost, and instrument‐free SARS‐CoV‐2 diagnostics in clinical samples.

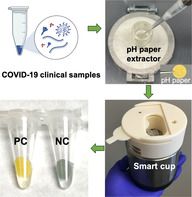

## 1 Introduction

In recent decades, public health has been challenged by infectious diseases caused by various viruses such as human immunodeficiency virus (HIV),^[^ 1, 2 ^]^ severe acute respiratory syndrome coronavirus (SARS‐CoV),^[^ ^3^ ^]^ influenza A virus subtypes H1N1^[^ ^4^ ^]^ and H7N9,^[^ ^5^ ^]^ Middle East respiratory syndrome coronavirus (MERS‐CoV),^[^ ^6^ ^]^ Zika virus,^[^ ^7^ ^]^ Ebola virus,^[^ ^8^ ^]^ and the currently emerging severe acute respiratory syndrome coronavirus 2 (SARS‐CoV‐2).^[^ ^9^ ^]^ Due to economic globalization, these public health challenges can easily trigger epidemic or even pandemic diseases.^[^ 10, 11 ^]^ As of now, the ongoing coronavirus disease 2019 (COVID‐19) pandemic resulting from SARS‐CoV‐2 has led to millions of deaths worldwide. Although a large‐scale COVID‐19 vaccination effort has been launched, rapid and streamlined SARS‐CoV‐2 diagnostics remain essential for early intervention and treatment of presymptomatic and asymptomatic COVID‐19 patients.^[^ 12, 13 ^]^

To combat the spread of COVID‐19, reverse transcription quantitative polymerase chain reaction (RT‐qPCR) is widely used as the gold standard for SARS‐CoV‐2 detection.^[^ 14, 15 ^]^ RT‐qPCR‐based RNA testing can provide rapid, sensitive, and specific SARS‐CoV‐2 diagnosis in comparison with other diagnostics methods such as antigen testing and serological testing of IgM and IgG.^[^ 16, 17 ^]^ However, RT‐qPCR methods greatly depend on well‐trained personnel, large and expensive detection instruments, and long reaction assay times, which are not amenable to rapid COVID‐19 testing onsite or in resource‐limited settings.^[^ ^18^ ^]^ As alternatives to RT‐qPCR for onsite COVID‐19 testing, researchers have developed different isothermal amplification assays such as reverse transcription loop‐mediated isothermal amplification (RT‐LAMP),^[^ ^19^ ^]^ reverse transcription recombinase polymerase amplification (RT‐RPA),^[^ ^20^ ^]^ and reverse transcription dual‐priming mediated isothermal amplification (RT‐DAMP).^[^ ^21^ ^]^

Currently, the primary materials employed for nucleic acid extraction are silica gel membranes and surface‐functionalized paramagnetic beads.^[^ 22, 23, 24 ^]^ These nucleic acid extraction systems require large benchtop centrifuges, magnetic rods, and well‐trained personnel, which are limiting and complicated for onsite pathogen detection. To eliminate the need for centrifugation and to simplify the extraction steps, researchers have developed paper materials, such as cellulose‐based Flinders Technology Associates (FTA) cards.^[^ 25, 26, 27 ^]^ However, FTA‐based nucleic acid extraction still requires relatively complex procedures such as punching, washing, and drying the cards from the sample areas. In addition, FTA cards are only used to archive nucleic acids, but are not able to mediate visual detection as an indicator. Thus, new functional materials are needed that can undertake both nucleic acid extraction and visual detection for onsite rapid diagnostics.

**Figure 1:**
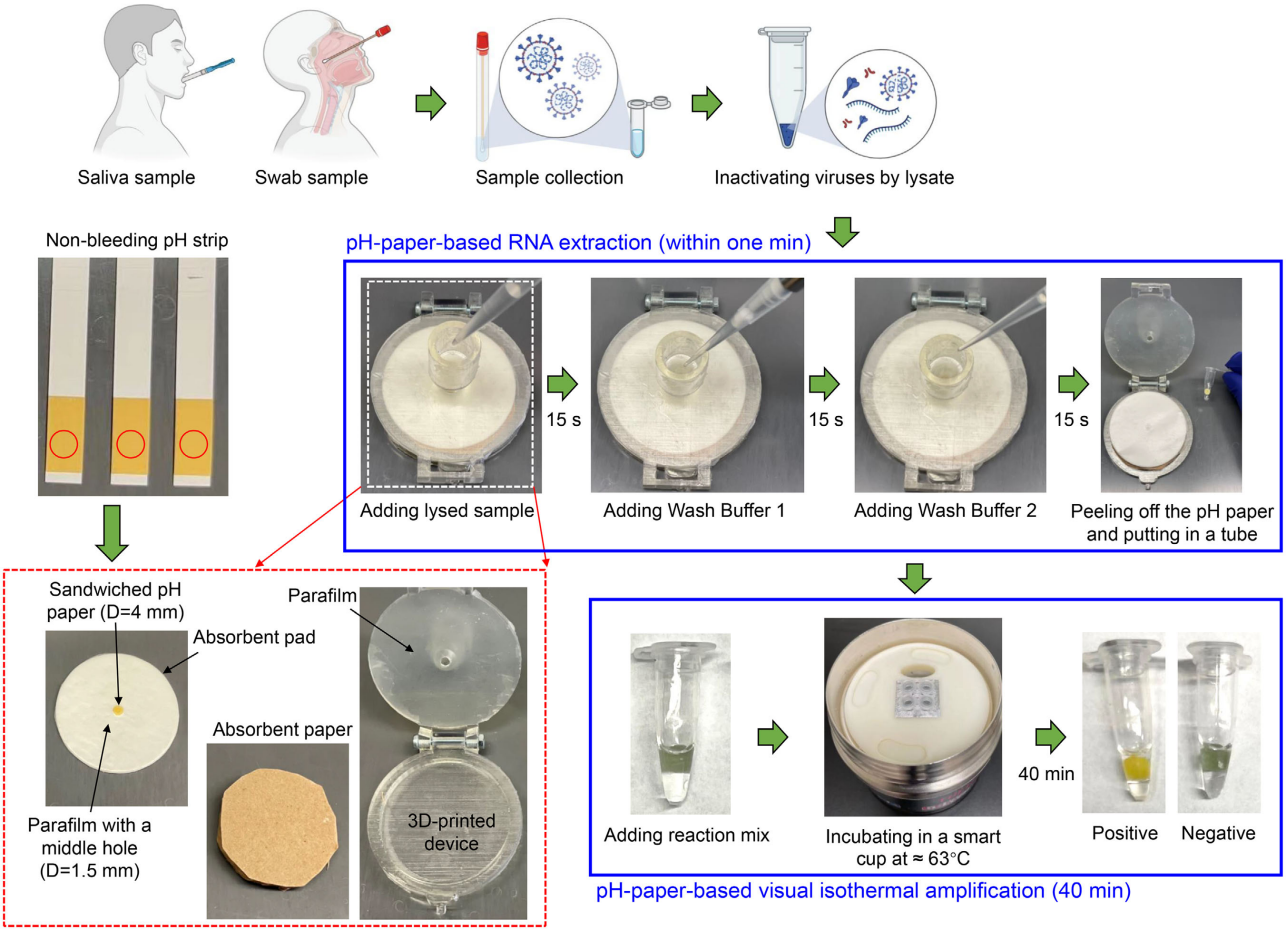
Schematic of instrument‐free SARS‐CoV‐2 detection enabled by pH‐paper‐based RNA extraction and visual detection (termed pH‐EVD).

In this study, inspired by pH test strips, we employed nonbleeding pH paper as a functional material to achieve both RNA extraction and visual isothermal amplification detection. The nonbleeding pH paper can provide a random matrix of cellulose fibers to extract and purify RNA from lysed clinical samples while avoiding contamination and inhibition of the subsequent isothermal amplification detection. After RNA extraction, the pH paper is compatible with the isothermal amplification reaction to mediate visual detection without the addition of any dyes. Accordingly, we developed a pH‐paper‐based extraction and visual detection (termed pH‐EVD) method for rapid, instrument‐free SARS‐CoV‐2 detection (**Figure** **1**). Through harnessing a chemically heated incubator called a smart cup, we clinically validated our pH‐EVD‐based instrument‐free SARS‐CoV‐2 detection on 30 clinical nasopharyngeal (NP) swab samples and 33 saliva samples spiked with RNA extracts from clinical NP swabs. Using the new function of nonbleeding pH paper, our pH‐EVD‐based SARS‐CoV‐2 detection offers a simple, rapid, reliable, low‐cost, and instrument‐free approach for onsite diagnostics of COVID‐19 or other infectious diseases.

## 2 Results and Discussion

### 2.1 Self‐Powered pH‐Paper Extraction System for Rapid Nucleic Acid Extraction

**Figure 2:**
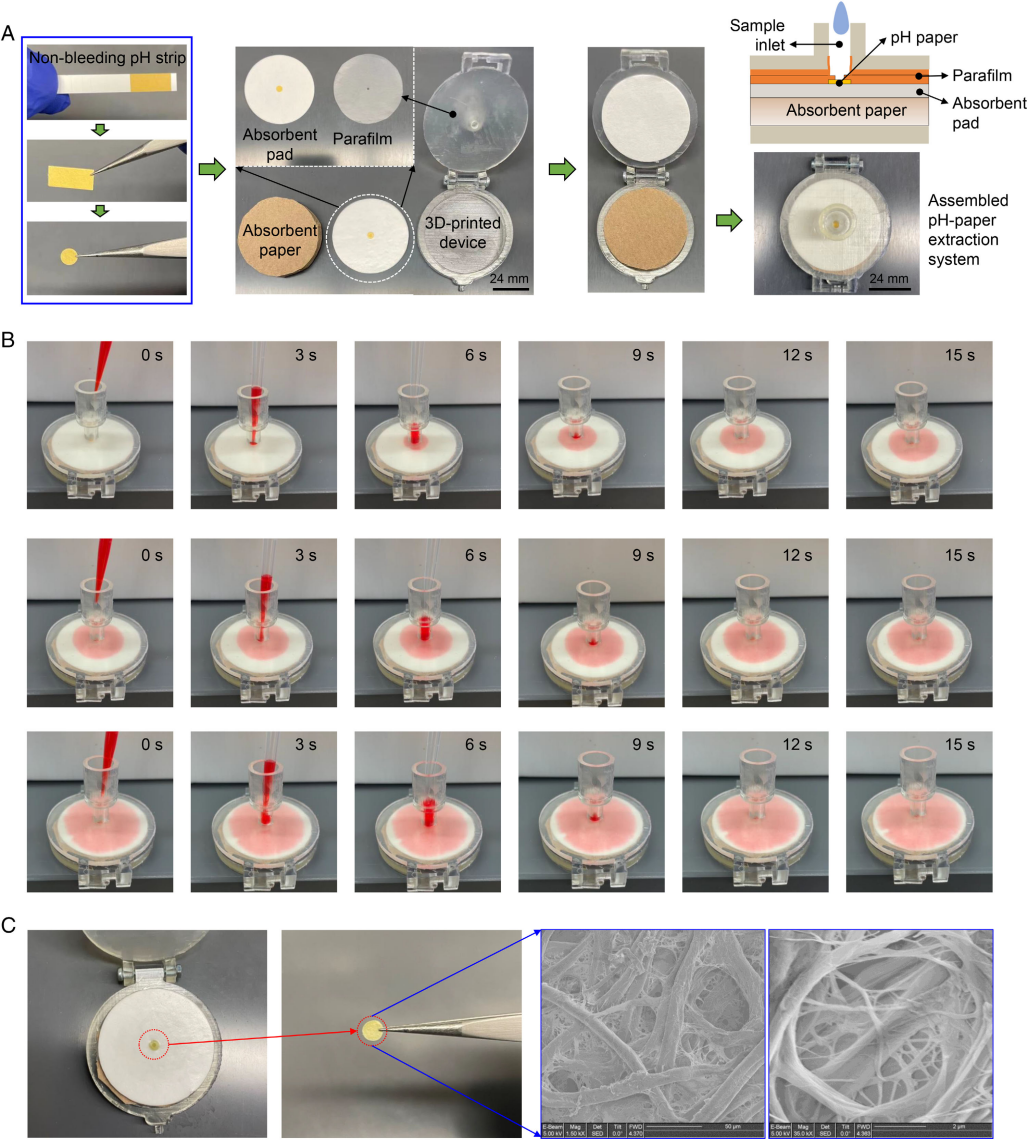
Development and characterization of the pH‐paper extraction system for rapid, instrument‐free nucleic acid preparation. A) Assembly of the pH‐paper extraction system in a 3D‐printed clamshell device. B) Time course of liquid transportation during three sequential liquid loadings. Red dye was added for visual purposes. C) Example SEM images of nonbleeding pH paper.

To achieve sensitive nucleic acid‐based molecular diagnostics, it is critical to extract high‐quality nucleic acids from raw samples. Here, we developed a self‐powered pH‐paper‐based extraction method for nucleic acid preparation (**Figure** **2**A), enabling instrument‐free nucleic acid purification and visual detection. By taking advantage of the nucleic acid binding properties of cellulose matrix,^[^ ^28^ ^]^ nonbleeding pH paper made of cellulose is, for the first time, used to extract nucleic acids from biological samples. Unlike common pH paper, the covalently bound indicator dyes in the nonbleeding pH paper can prevent from bleeding and contaminating the samples.^[^ ^29^ ^]^ Further, the pH indicator dyes in the paper are able to mediate visual isothermal amplification detection without supplying additional dyes. Thus, nonbleeding pH paper can serve as a new functional material to undertake both nucleic acid extraction and visual detection. As shown in Figure 2A, we first obtained a pH paper disc from a commercially available nonbleeding pH strip (pH 6.5–10.0). Next, we sandwiched the pH paper disc between a parafilm layer and an absorbent pad/paper stack. To assemble a self‐powered pH‐paper extraction system, we fabricated a clamshell device (Figure S1, Supporting Information) by 3D printing to package the parafilm layer/pH paper disc/absorbent stack (Figure 2A).

In the pH‐paper extraction system, the hydrophilic absorbent pad/paper stack provides capillary force for liquid transportation and waste collection, eliminating the need for an external pump or centrifuge. To mock the conventional nucleic acid extraction process (e.g., QIAamp Viral RNA Mini Kit), we added three liquid samples with food dye into our pH‐paper extraction system. As shown in Figure 2B, the entire nucleic acid preparation process can be completed within 45 s, which is over 20 times faster than conventional nucleic acid extraction methods by centrifugation. Alternatively, the pipettes can be simply replaced with plastic squeezable liquid dropper bottles, enabling simple, instrument‐free RNA extraction by minimally trained personnel at home, at drive‐through testing sites, or in the doctor's office. Figure 2C shows scanning electron microscope (SEM) images of nonbleeding pH paper. The nucleic acids bound on the cellulose fibers of the pH paper are extracted and purified. After immersing it in isothermal amplification reaction solution, the bound nucleic acids in the pH paper are released to initiate the visual detection as the templates at 63 °C. Thus, the pH‐paper extraction system provides a simple, rapid, self‐powered nucleic acid sample preparation approach without the need for any equipment (e.g., a centrifuge machine).

### 2.2 Development of pH‐Paper‐Based Visual Isothermal Amplification Detection Approach

**Figure 3:**
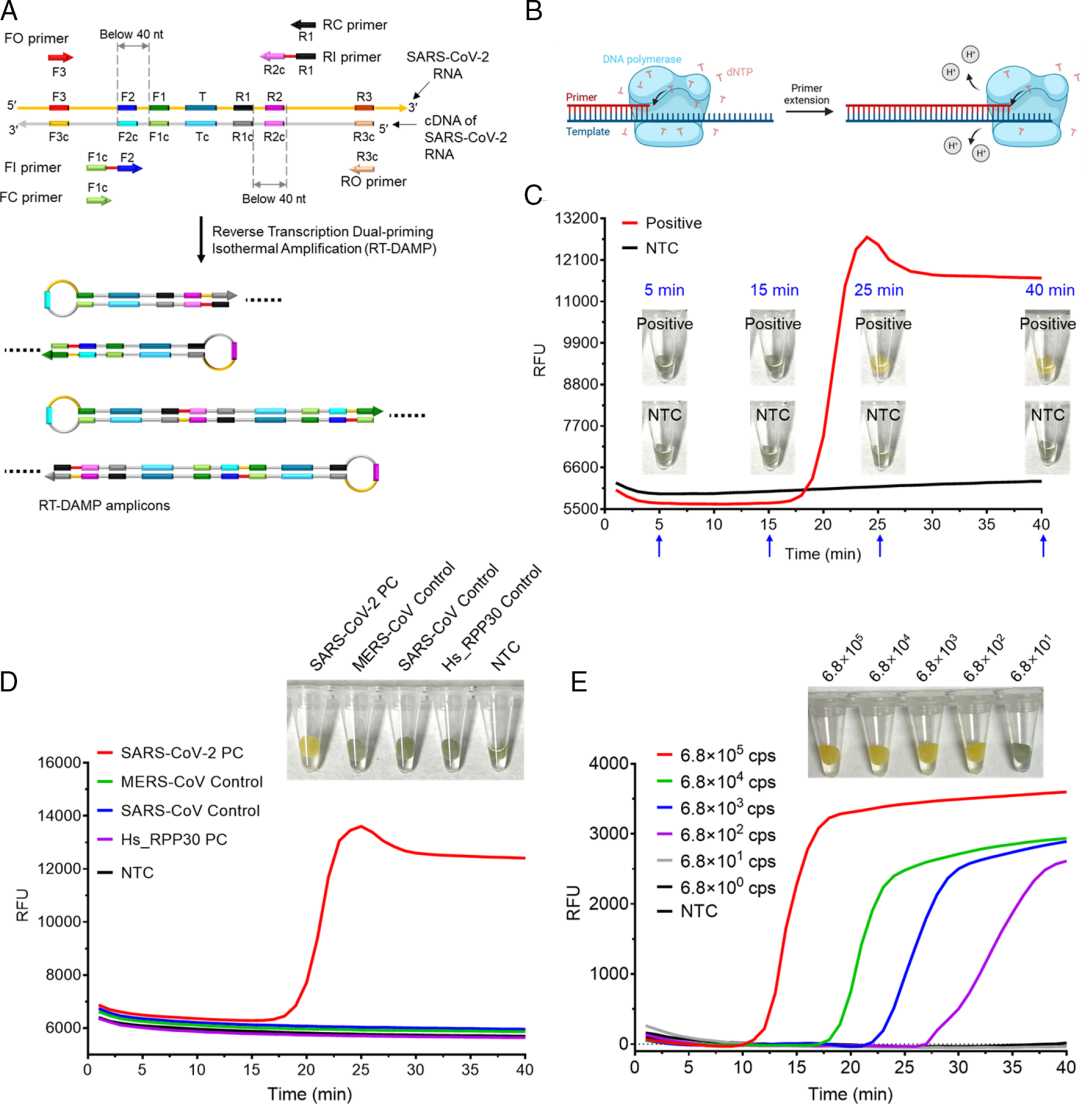
Visual detection of isothermal amplification by direct insertion of nonbleeding pH paper disc into the reaction solutions. A) Schematic of RT‐DAMP. B) Hydrogen ions produced during primer extension in isothermal amplification. C) Time course of the pH paper color change and the EvaGreen‐based real‐time fluorescence detection. Positive, reactions with 6.8 × 10^4^ copies of SARS‐CoV‐2 RNA. NTC, reactions without any template. D) Specificities of pH‐paper‐based visual detection and real‐time fluorescence detection. SARS‐CoV‐2 PC, SARS‐CoV control, MERS‐CoV control, and Hs_RPP30 PC are commercial plasmids with corresponding gene sequences (Integrated DNA Technologies). E) Sensitivities of pH‐paper‐based visual detection and real‐time fluorescence detection. Various copies of commercial SARS‐CoV‐2 RNA control from Twist Bioscience were used. The visual detection was conducted with a 40 min incubation. Each experiment involving real‐time fluorescence detection and tube‐based visual detection was repeated three times.

With the innate capability of sensing pH change, nonbleeding pH paper can mediate visual isothermal amplification detection. In this study, we used RT‐DAMP, an isothermal amplification method previously developed in our lab.^[^ ^30^ ^]^ As shown in **Figure** **3**A, six RT‐DAMP primers specifically recognize seven distinct sites in the target RNA and its cDNA, and the main amplicons are multiple double‐stranded DNAs with closed loops. In a nonbuffered RT‐DAMP solution, hydrogen ions (H^+^) are generated and accumulate to decrease the pH,^[^ ^31^ ^]^ which is attributed to the incorporation of nucleotides during primer extension by DNA polymerase (Figure 3B). To prepare the nonbuffered solution, Tris‐HCl is typically removed to permit the pH change, while KOH is used to tune an alkaline condition (pH around 8.5) for the initiation of amplification.^[^ 32, 33, 34 ^]^ Therefore, by directly inserting pH paper disc into the RT‐DAMP solution, we successfully developed a simple visual isothermal amplification detection. As shown in Figure 3C, pH paper clearly presents as yellow in positive reactions with targets in as short as 25 min, and stays a green‐brown color in no‐template control (NTC) reactions. Further, the colorimetric results are in accordance with the results of real‐time fluorescence detection.

Next, we assessed the specificity of pH‐paper‐based visual isothermal amplification. As shown in Figure 3D, after a 40 min incubation, only the SARS‐CoV‐2 positive control leads to a yellow color reaction, validating high specificity of the visual assay. In addition, we assessed the sensitivity by testing synthetic SARS‐CoV‐2 RNA controls from Twist Bioscience. As depicted in Figure 3E, pH‐paper‐based visual RT‐DAMP can detect down to 680 copies of SARS‐CoV‐2 RNA within 40 min, showing similar sensitivity as real‐time fluorescence detection of RT‐DAMP. Thus, pH‐paper‐based visual isothermal amplification has high specificity and sensitivity for SARS‐CoV‐2 detection.

### 2.3 Optimization of pH‐Paper‐Based RNA Extraction

**Figure 4:**
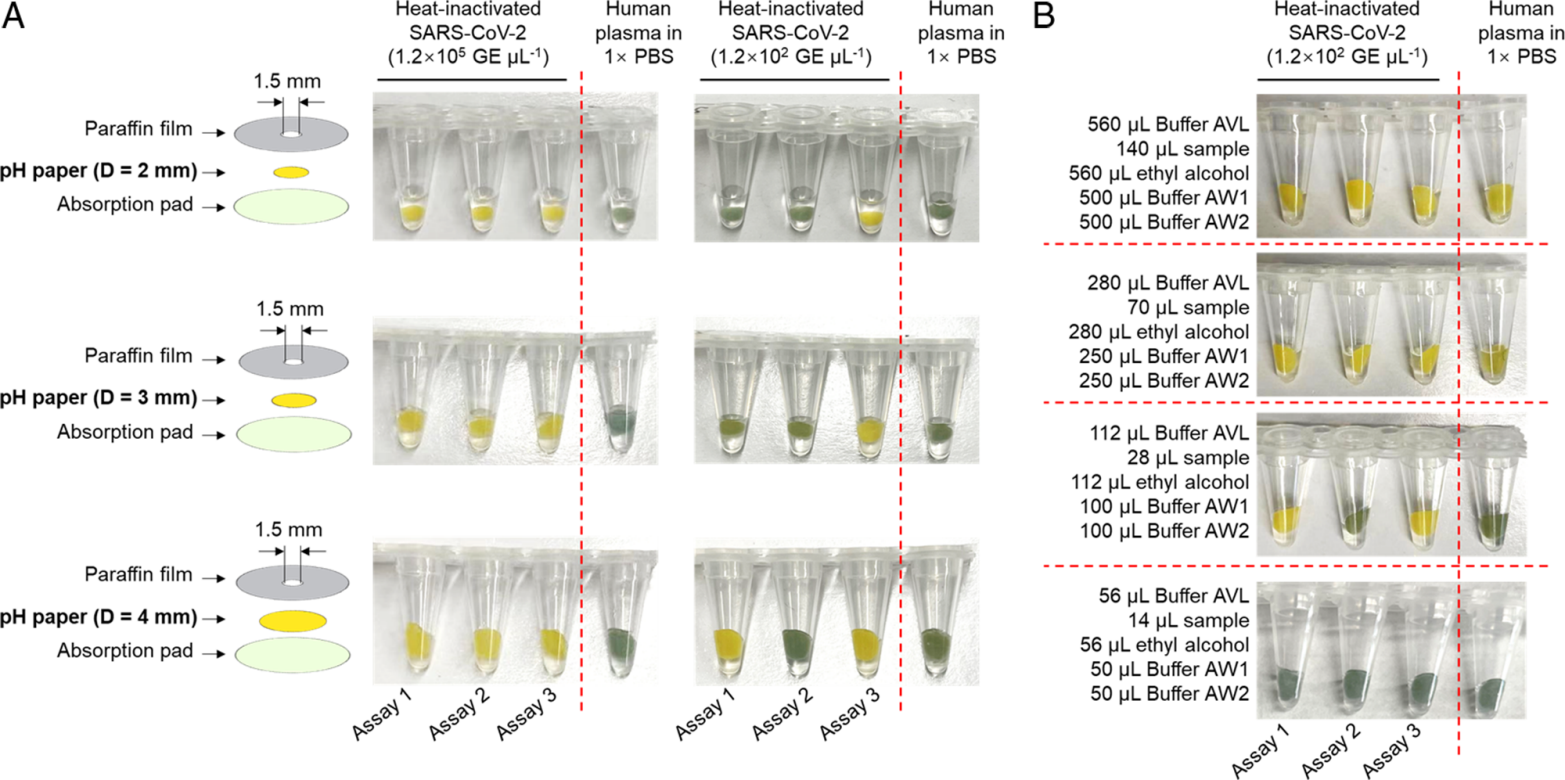
Optimization of pH‐paper‐based RNA extraction for visual isothermal amplification detection. A) Effect of various diameters of pH paper disc on extraction performance. In this experiment, 28 μL sample, 112 μL Buffer AVL, 112 μL absolute ethyl alcohol, 112 μL Buffer AW1, and 112 μL Buffer AW2 from the commercial QIAamp Viral RNA Mini Kit (QIAGEN) were used. Buffer AVL is a lysis buffer. Buffer AW1 and AW2 are two wash buffers. B) Effect of varying the amounts of the extraction reagents on extraction performance. The ratio of Buffer AVL, absolute ethyl alcohol, Buffer AW1, and Buffer AW2 strictly follows the kit's instructions. Heat‐inactivated SARS‐CoV‐2 was provided by BEI Resources. Three replicates (Assays 1–3) were set up for each test with the heat‐inactivated SARS‐CoV‐2.

Our self‐powered pH‐paper RNA extraction system employs the commercial RNA extraction reagents provided by the QIAamp Viral RNA Mini Kit to extract viral RNA from clinical samples. To improve the detection performance, we optimized the diameter of the pH paper disc and the amounts of extraction reagents, followed by visual detection. As shown in **Figure** **4**A, we found that the larger the diameter of the pH paper disc, the higher is the sensitivity of visual detection. This improvement is likely due to the enlarged filter area provided by the pH paper disc. The maximum pH paper disc diameter we used was 4 mm, as diameters above 4 mm cannot be fully immersed by the reaction solution, disrupting the visual detection.

After selecting a 4 mm‐diameter pH paper disc, we investigated various amounts of extraction reagents at the same ratio of volume recommended in the kit's instructions. Figure 4B shows that the combination of 28 μL sample, 112 μL Buffer AVL, 112 μL absolute ethyl alcohol, 112 μL Buffer AW1, and 112 μL Buffer AW2 gives the best performance. We also found that adding more extraction reagents leads to false positives, likely because it brings about excessive residues on the pH paper and disturbs the initial reaction solution pH.

### 2.4 Performance of pH‐EVD Method for SARS‐CoV‐2 Detection

**Figure 5:**
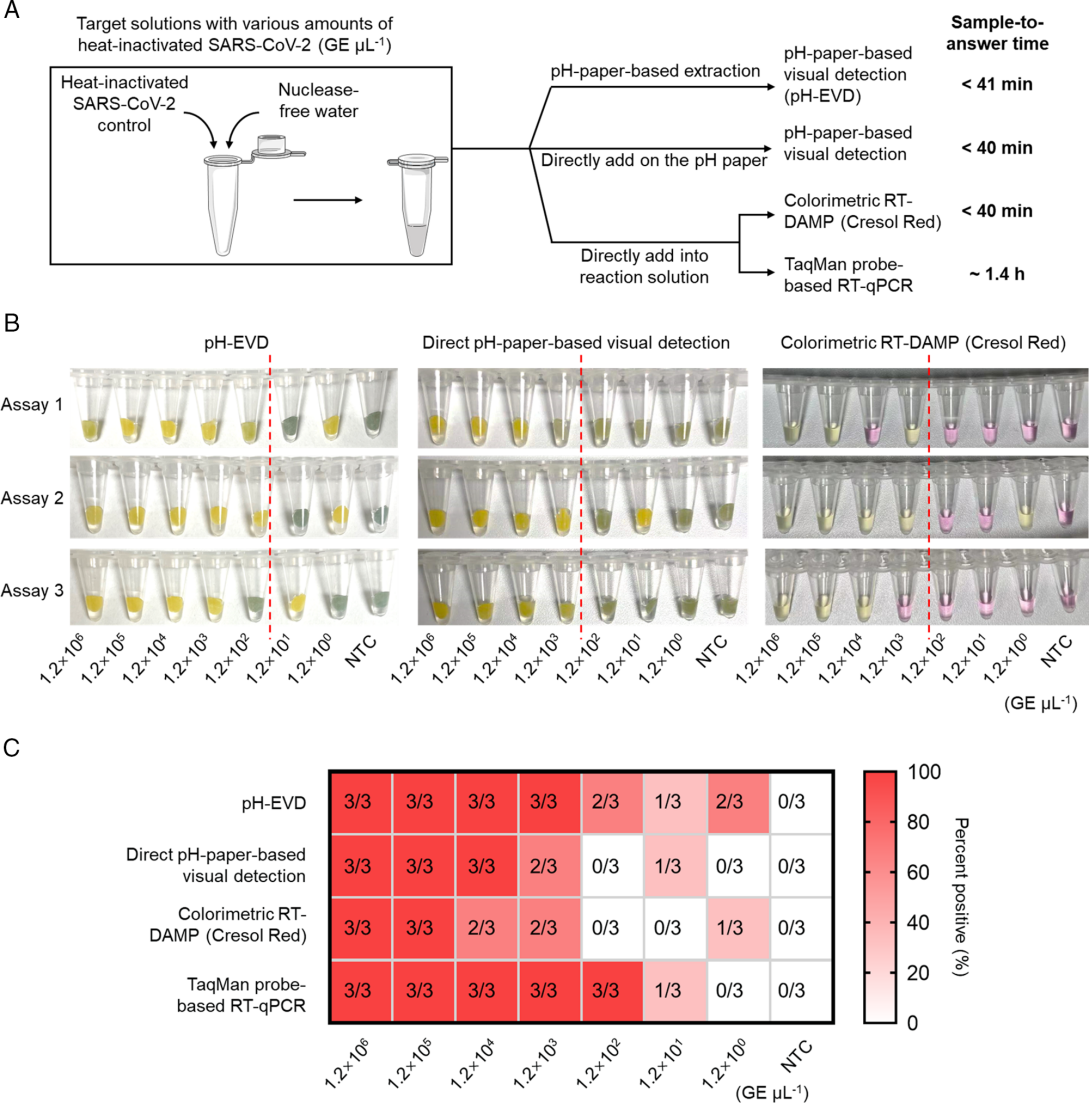
Performance of the pH‐EVD method using various amounts of heat‐inactivated SARS‐CoV‐2. A) Procedures of the pH‐EVD method and the parallel assays including direct pH‐paper‐based visual detection, extraction‐free colorimetric RT‐DAMP using cresol red, and extraction‐free TaqMan probe‐based RT‐qPCR. B) Comparison of visual detection results. C) Percent positives for various assays. All of the visual detections were incubated for 40 min. GE, genome equivalents. NTC, solutions without any heat‐inactivated SARS‐CoV‐2. Three independent experiments (Assays 1–3) were set up for each test group.

To evaluate the detection performance of the pH‐EVD method, we prepared a panel of heat‐inactivated SARS‐CoV‐2 controls (BEI Resources) at a serial tenfold dilution from 1.2 × 10^6^ to 1.2 × 10^0^ GE μL^−1^. For comparison purposes, we performed direct pH‐paper‐based visual detection without RNA extraction, extraction‐free colorimetric RT‐DAMP using cresol red dye,^[^ ^35^ ^]^ and extraction‐free TaqMan probe‐based RT‐qPCR in parallel experiments (**Figure** **5**A).

As shown in Figure 5B, the pH‐EVD method showed 100% (3/3) detection of 1.2 × 10^3^ GE μL^−1^ heat‐inactivated SARS‐CoV‐2 in the target solutions, and 66.7% (2/3), 33.3% (1/3), and 66.7% (2/3) detection of 1.2 × 10^2^, 1.2 × 10^1^, and 1.2 × 10^0^ GE μL^−1^, respectively. However, when we removed the pH‐paper‐based RNA extraction step, we only achieved 66.7% (2/3) detection of 1.2 × 10^3^ GE μL^−1^ for both pH‐paper‐based and dye‐based visual detections. In addition, we found that the color change by the pH‐EVD method is more significant than that without extraction, demonstrating that unpurified samples can potentially interfere with the initial pH of reaction solutions and weaken the performance.

In contrast, extraction‐free RT‐qPCR showed higher performance with 100% (3/3) detection of 1.2 × 10^2^ GE μL^−1^ SARS‐CoV‐2 (Figure 5C). The average quantification cycle (*C*_q_) value was 35.96 (Figure S2, Supporting Information), which was close to the cutoff suggested by the CDC (*C*_q_ = 40).^[^ ^36^ ^]^ In addition, the extraction‐free RT‐qPCR assay requires a long testing time (≈1.4 h) from sample to answer (Figure 5A) and greatly depends on large detection instruments. Thus, the pH‐EVD method with a less than 41 min sample‐to‐answer time is more advantageous for point‐of‐care testing.

**Figure 6:**
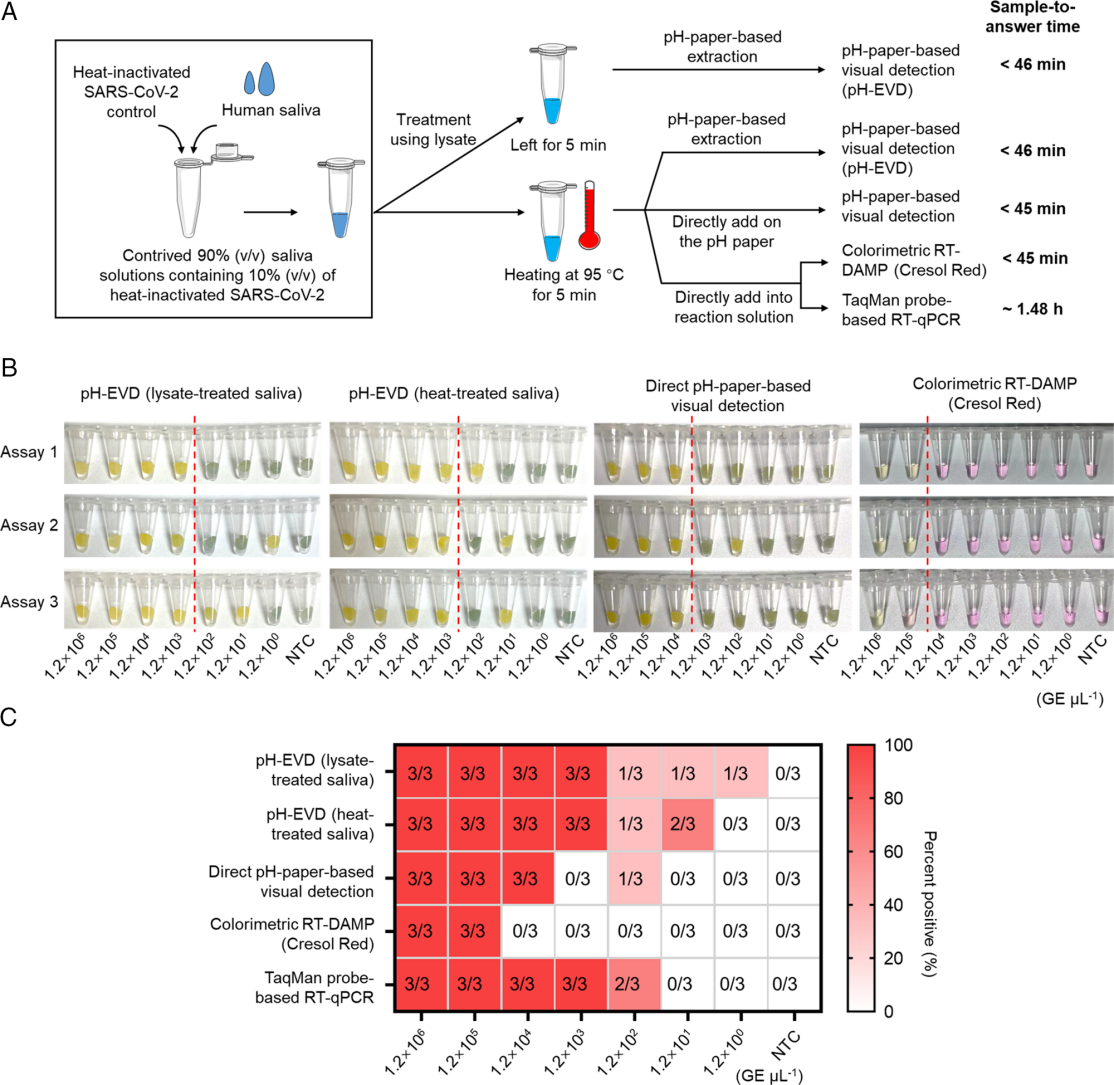
Performance of the pH‐EVD method on detecting various amounts of heat‐inactivated SARS‐CoV‐2 spiked in human saliva. A) Procedures of the pH‐EVD method with lysate and heat‐treatment of saliva, and the parallel assays with heat‐treated saliva for direct pH‐paper‐based visual detection, extraction‐free colorimetric RT‐DAMP using cresol red, and extraction‐free TaqMan probe‐based RT‐qPCR. B) Comparison of visual detection results. C) Percent positives for various assays. All the visual detections were incubated for 40 min. GE, genome equivalents. NTC, solutions without any heat‐inactivated SARS‐CoV‐2. Three independent experiments (Assays 1–3) were set up for each test group.

Next, we contrived 90% v/v saliva solutions by spiking 10% v/v of the same SARS‐CoV‐2 controls to further demonstrate the performance of our pH‐EVD method. Prior to pH‐paper‐based RNA extraction or direct addition, we treated the contrived saliva solutions with lysis buffer (Buffer AVL) or heated them at 95 °C for 5 min (**Figure** **6**A). In less than a 46 min sample‐to‐answer time, the pH‐EVD method could detect 100% (3/3) of 1.2 × 10^3^ GE μL^−1^ SARS‐CoV‐2 from saliva solutions treated with both lysate and heat, showing the same sensitivity as that of assaying serial dilutions of heat‐inactivated SARS‐CoV‐2 control (Figure 5B and 6B). However, without RNA extraction, direct pH‐paper‐based visual detection and colorimetric RT‐DAMP using cresol red demonstrated a lower sensitivity, only showing consistent detection of 1.2 × 10^4^ and 1.2 × 10^5^ GE μL^−1^ SARS‐CoV‐2, respectively. This result suggests that heat‐treated saliva can inhibit extraction‐free visual isothermal amplification detection.^[^ ^37^ ^]^ While we found that RT‐qPCR can consistently detect 1.2 × 10^3^ GE μL^−1^ SARS‐CoV‐2 in saliva, the average *C*_q_ value was 36.77 (Figure S3, Supporting Information), higher than that without saliva (average *C*_q_ value of 32.12, Figure S2, Supporting Information). In addition, the RT‐qPCR assay showed decreased sensitivity from 1.2 × 10^2^ to 1.2 × 10^3^ GE μL^−1^. Thus, the performance of RT‐qPCR is also influenced by heat‐treated saliva. Collectively, by taking advantage of pH‐paper‐based nucleic acid extraction, the pH‐EVD method does not only improve the detection sensitivity, but also increases detection signals by minimizing the interference of raw saliva samples.

### 2.5 Clinical Validation of pH‐EVD‐Enabled Instrument‐Free SARS‐CoV‐2 Detection

To demonstrate the feasibility of instrument‐free SARS‐CoV‐2 detection in clinical samples, we coupled the pH‐EVD method with our previously reported handheld smart cup platform.^[^ 34, 38 ^]^ In the smart cup platform (Figure S4A, Supporting Information), an exothermic chemical reaction triggered by water provides heat and a phase‐change material regulates the temperature at 60–65 °C for isothermal amplification. To adapt the reaction tubes for the pH‐EVD method, we simply replaced the heat sink in our previous smart cup with a 3D‐printed metal tube holder (Figure S4B, Supporting Information).

**Figure 7:**
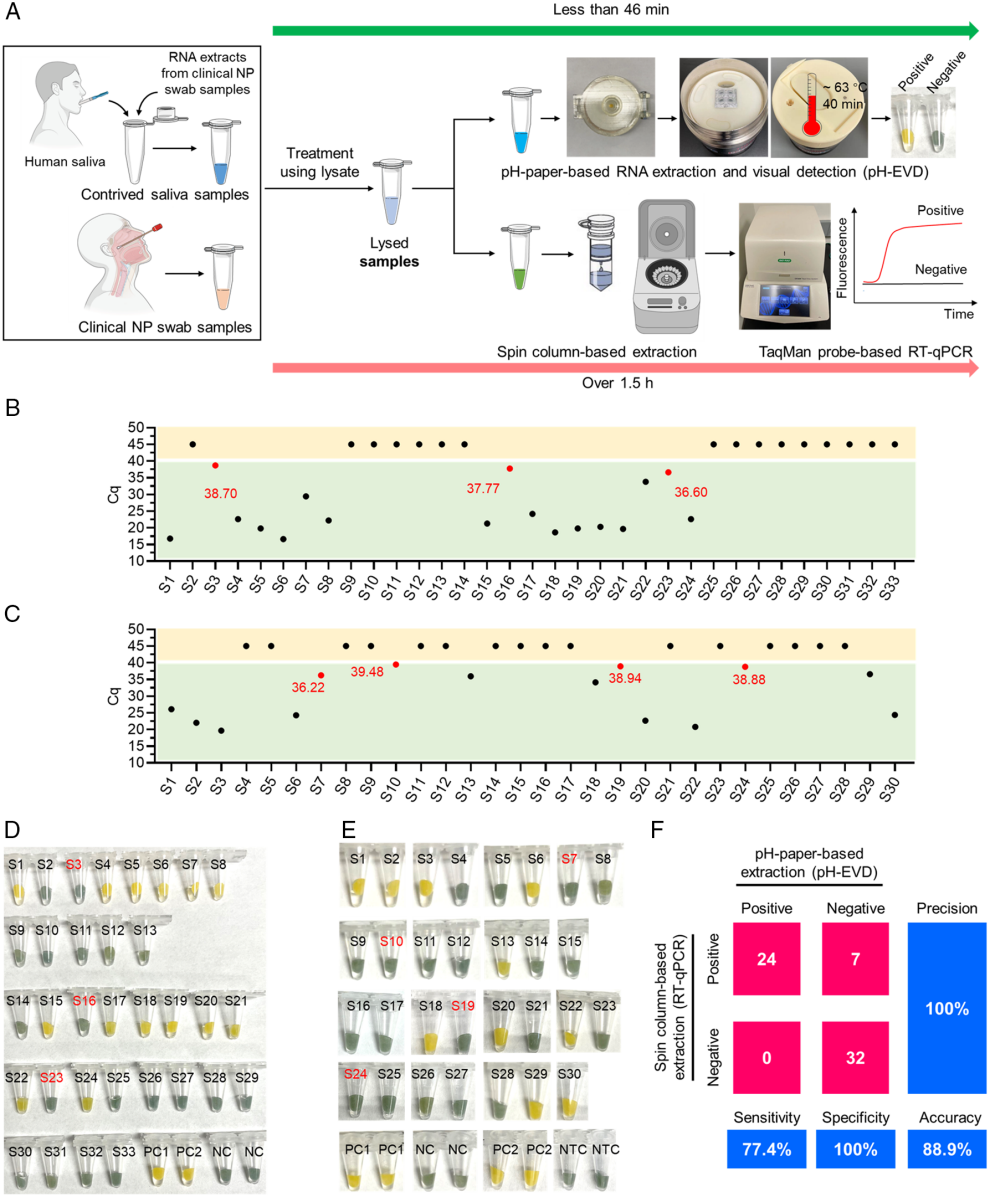
Clinical validation of rapid instrument‐free SARS‐CoV‐2 detection by the pH‐EVD method. A) Procedures of the instrument‐free detection method and the routine RT‐qPCR assay for testing clinical NP samples and contrived saliva samples. B,C) *C*_q_ values of the 33 saliva samples and 30 clinical NP samples by RT‐qPCR following spin column‐based extraction, respectively. D,E) Visual detection results of the instrument‐free pH‐EVD method for the saliva and NP samples, respectively. F) Confusion matrix describing the overall performances of the two assays between positive and negative samples. The cutoff of *C*_q_ was 40. The spin column‐based extraction and RT‐qPCR are considered as the standards. The samples marked with red were tested to be negative by the pH‐EVD method, while positive by RT‐qPCR. Each experiment was repeated three times.

As shown in **Figure** **7**A, we randomly selected clinical NP swab samples and treated using them with lysis buffer. Next, we subjected the lysed samples to the pH‐EVD assay. For comparison, we used a routine SARS‐CoV‐2 detection approach of fluorescence‐based real‐time RT‐qPCR following spin column‐based RNA extraction. Due to lack of access to clinical saliva samples, we contrived them by spiking clinical RNA extracts from NP samples into human saliva. The instrument‐free SARS‐CoV‐2 detection method was able to complete the entire assay from lysing samples to getting results within 46 min, suitable for onsite diagnostics. By contrast, the routine RT‐qPCR assay requires more than 1.5 h and must be conducted in central laboratories with benchtop equipment (e.g., a centrifuge machine, PCR machine).

Figure 7B,C displays the *C*_q_ values for contrived saliva samples (*n* = 33) and clinical NP swab samples (*n* = 30) by RT‐qPCR, respectively. As per the cutoff (*C*_q_ = 40) suggested by the CDC,^[^ ^36^ ^]^ 31 samples were tested to be positive (17 for saliva and 14 for the NP swab) and 32 samples were negative (16 for both saliva and the NP swab). Figure 7D,E shows the visual detection results of the pH‐EVD method on testing the saliva and NP swab samples, respectively. By contrast, most of the pH‐EVD methods’ results conform with those of RT‐qPCR, except the saliva samples of 3, 16, and 23, and the NP swabs of 7, 10, 19, and 24. For these seven samples, the *C*_q_ values ranged from 36.22 to 39.48, very close to the cutoff, therefore considering them to be very weak positives. With spin column‐based extraction and RT‐qPCR set as the standards, the confusion matrix indicates 77.4% sensitivity, 100% specificity, and 100% precision for our pH‐EVD assay (Figure 7F). The reduced sensitivity is likely attributed to a small volume of samples (28 μL) supplied for pH‐paper‐based RNA extraction, compared to that (140 μL) for spin column‐based extraction. However, if focusing on the samples with the cutoff of *C*_q_ = 35, our pH‐EVD platform shows 100% sensitivity and 100% specificity (Figure S5, Supporting Information). Therefore, as a simple, low‐cost, instrument‐free diagnostic approach, our pH‐EVD‐based method provides a promising diagnostic alternative for rapid SARS‐CoV‐2 diagnostics as a preliminary screening tool.

## 3 Conclusion

In summary, we developed a rapid, instrument‐free SARS‐CoV‐2 detection method enabled by pH‐EVD for onsite testing. To the best of our knowledge, this is the first report of employing nonbleeding pH paper to extract RNA within 1 min and then directly mediate dye‐free visual isothermal amplification detection. Toward onsite COVID‐19 diagnostics, our pH‐EVD‐based SARS‐CoV‐2 detection method has several advantages over the standard RT‐qPCR assay. First, the entire detection procedure is simple and independent of expensive electric instruments. Second, it is rapid and straightforward, requiring less than 46 min from sample to result. Third, it performs robust detection, even on saliva samples. Fourth, it is low cost, costing about $2.87 per test including RNA extraction and visual detection when using all commercial materials and reagents (Figure S6, Supporting Information). Bulk discount will further reduce the cost when scaled up for mass production. Last, our proposed pH‐EVD platform is more advantageous over current paper‐based technologies due to simplicity, rapidity, low cost, and electricity‐free characteristics (Table S2, Supporting Information). Therefore, we envision that the instrument‐free pH‐EVD detection platform can be applied to streamline rapid detection of COVID‐19 or other infectious diseases.

## 4 Experimental Section

4.1

4.1.1

##### Materials

MColorpHast pH‐indicator strips (nonbleeding; Cat. No. 1.09543.0001) for pH 6.5–10.0, round absorbent pad (AP1004700), betaine (5.0 m), (NH_4_)_2_SO_4_, Tween 20, KCl, cresol red, and KOH were purchased from MilliporeSigma (Burlington, MA). Parafilm M laboratory wrapping film and absolute ethyl alcohol were purchased from Fisher Scientific (East Greenwich, RI). EvaGreen dye (20×) was purchased from Biotium (Fremont, CA). The QIAamp Viral RNA Mini Kit was purchased from Qiagen (Hilden, Germany). Mg_2_SO_4_ (100 mM), dNTP mix (10 mM of each), ET SSB (500 μg mL^−1^), Bst 2.0 WarmStart DNA polymerase (Bst 2.0 WS; 8000 U mL^−1^), WarmStart RTx reverse transcriptase (WS RTx; 15 000 U mL^−1^), and nuclease‐free water were purchased from New England BioLabs (Ipswich, MA). The GoTaq Probe 1‐Step RT‐qPCR kit was purchased from Promega (Madison, WI). Heat‐inactivated SARS‐CoV‐2 (isolate USA‐WA1/2020) control was obtained from BEI Resources (Manassas, VA). Twist synthetic SARS‐CoV‐2 RNA control was purchased from Twist Bioscience (South San Francisco, CA). SARS‐CoV‐2N positive control (SARS‐CoV‐2 _PC), SARS‐CoV control, MERS‐CoV control, human RPP30 gene control (Hs_RPP30_PC), and all the primers and probes for both the CDC‐released SARS‐CoV‐2 N1 RT‐qPCR assay and pH‐EVD were purchased from Integrated DNA Technologies (Coralville, IA). Pooled human saliva (5.0 mL) was purchased from Innovative Research, Inc. (Novi, MI). A total of 30 clinical NP swab samples and 33 RNA extracts from the clinical NP swab samples were handled in compliance with ethical regulations and the approval of the Institutional Review Board of the University of Connecticut Health Center (protocol #: P61067).

##### RNA Extraction

RNA extraction from heat‐inactivated SARS‐CoV‐2 control, contrived, and clinical samples were conducted by using pH‐paper‐based extraction or spin column‐based extraction. For pH‐paper‐based extraction, the pH paper disc was first sandwiched between a parafilm layer and an absorbent pad. Next, the sandwiched pH paper disc was placed in a 3D‐printed device filled with absorbent paper. After sample collection, a 28 μL aliquot of the sample was mixed with 112 μL Buffer AVL and 112 μL absolute ethyl alcohol before loading into the device. Sixteen seconds later, 100 μL Wash Buffer 1 was added for the first wash. Afterward (about 15 s), 100 μL Wash Buffer 2 was added for the second wash. Subsequently, the pH paper disc was peeled off and added into reaction tubes. The Buffer AVL, Wash Buffer 1, and Wash Buffer 2 were all obtained from the QIAamp Viral RNA Mini Kit. For spin column‐based extraction, the procedure was strictly according to the kit's instructions. Briefly, a 140 μL aliquot of sample was mixed with 560 μL Buffer AVL and 560 μL absolute ethyl alcohol, prior to addition into the spin column, followed by two washes using 500 μL Wash Buffer 1 and 500 μL Wash Buffer 2. During the extraction, large benchtop centrifuge was used to filter the samples. The RNA was finally eluted by using nuclease‐free water instead of Buffer AVE. All of the RNA extracts were aliquoted and kept at ‐80 °C until use.

##### pH‐Paper‐Based Visual Isothermal Amplification Detection

In this study, RT‐DAMP was used as the isothermal amplification method. Table S1, Supporting Information, shows the sequence information of the six RT‐DAMP primers including FO, RO, FI, RI, FC, and RC. These primers were designed according to the principles previously reported by our lab.^[^ ^30^ ^]^ To develop pH‐paper‐based visual detection, a nonbuffered solution (2×) was first prepared by combining 20 mM (NH_4_)_2_SO_4_, 0.2% v/v Tween 20, 100 mM KCl, 8 mM KOH, 2.8 mM dNTP, and 16 mM MgSO_4_ in a total 500 μL volume. A typical 20 μL pH‐paper‐based visual isothermal amplification reaction included 1 × nonbuffered solution, 0.2 μM FO, 0.2 μM RO, 1.6 μM FI, 1.6 μM RI, 1.6 μM FC, 1.6 μM RC, 0.3 U μL^−1^ WS RTx, 2.5 ng μL^−1^ ET SSB, 0.2 m betaine, 1.2 U μL^−1^ Bst 2.0 WS, and the inserted pH paper disc. For real‐time fluorescence detection without the pH paper disc, 0.8 × EvaGreen and 2.5 μL template solution were added. The prepared reactions were then subjected to incubation at 63 °C for 40 min in the CFX96 Touch Real‐Time PCR Detection System (Bio‐Rad, USA) or the handheld smart cup.

##### Instrument‐Free SARS‐CoV‐2 Detection

Thirty deidentified clinical NP swab samples were first treated for 10 min using the lysis buffer (Buffer AVL) before RNA extraction in the clinical microbiology laboratory at UConn Health. Then, the lysed samples were placed in the 3D‐printed device for pH‐paper extraction. After less than a 1 min extraction, the pH paper disc was peeled off and directly inserted into the isothermal amplification solution described above, followed by a 40 min incubation in the smart cup. The result was immediately read based on the color change of the pH paper disc. Thus, the instrument‐free SARS‐CoV‐2 detection could be completed within 46 min from sample to answer. The 33 contrived saliva samples were prepared by spiking 33 RNA extracts from clinical NP samples into human saliva at a volume ratio of 1:9. These saliva samples were then subjected to similar processes as those for the clinical NP swabs.

##### Real‐Time Fluorescence RT‐qPCR Assay

The RT‐qCPR assay by targeting the N gene to detect SARS‐CoV‐2 was conducted strictly according to the CDC‐released instructions titled CDC 2019‐Novel Coronavirus (2019‐nCoV) Real‐Time RT‐PCR Diagnostic Panel (https://www.fda.gov/media/134922/download). The GoTaq Probe 1‐Step RT‐qPCR kit recommend by the CDC was used to prepare the reaction mix. A typical 15 μL RT‐qPCR reaction included 1 × GoTaq Probe Master Mix, 0.5 μM nCOV_N1 forward primer, 0.5 μM nCOV_N1 reverse primer, 0.125 μM nCOV_N1 probe, 0.3 μL of the GoScript Reverse Transcriptase Mix, and 1.0 μL of the target solution. The thermal cycling program consisted of Stage 1 (2.0 min at 25 °C), Stage 2 (15.0 min at 50 °C), Stage 3 (2.0 min at 95 °C), and Stage 4 (45 cycles of 3.0 s at 95 °C and 30 s at 55 °C). The capture point of fluorescence was set at 55 °C in Stage 4. Real‐time fluorescence detection was carried out in the Bio‐Rad CFX96 Touch Real‐Time PCR Detection System.

##### Statistical Analysis

Each graph involving real‐time fluorescence detection or tube‐based visual detection is a representative of the similar results from three technical replicates. For performance comparison, three independent experiments (Assays 1–3) were set up. Statistical significances (the unpaired two‐tailed *t*‐test with the defined significance of *p*‐value < 0.05), heat maps with percent positive, and the confusion matrix of the test results were all analyzed by using the GraphPad Prism 8 software (version 8.0.1).

## Conflict of Interest

The authors declare no conflict of interest.

## Supporting information

Supplementary Material

## Data Availability

Research data are not shared.
