# Supplementary material for: pH‐EVD: A pH‐Paper‐Based Extraction and Visual Detection System for Instrument‐Free SARS‐CoV‐2 Diagnostics
Source: Adv Nanobiomed Res. 2021 Dec 7;2(2):2100101. doi: 10.1002/anbr.202100101 (PMC9011642; doi:10.1002/anbr.202100101)
Supplement: Supplementary file 1 — Supplementary Material [file ANBR-2-2100101-s001.pdf]

Supporting Information

pH-EVD: A pH-Paper-based Extraction and Visual Detection System for Instrument-free SARS-CoV-2 Diagnostics

Xiong Ding[1], Ziyue Li[1], Lori Avery[2], Enrique Ballesteros[2], Rohit Makol[1], and Changchun Liu[1]*

[1]Department of Biomedical Engineering, University of Connecticut Health Center, 263 Farmington Ave., Farmington, CT 06030, United States

[2]Department of Pathology and Laboratory Medicine, University of Connecticut Health Center, Farmington, CT 06030, United States

[*] **Corresponding author**

Dr. Changchun Liu

Department of Biomedical Engineering

University of Connecticut Health Center

263 Farmington Avenue

Farmington, CT 06030

Phone: (860)-679-2565

E-mail: chaliu@uchc.edu

1. Supporting Experimental Section

Contrived Saliva Samples Preparation: Due to lack of access to clinical saliva samples, we contrived the saliva samples by spiking heat-inactivated SARS-CoV-2 control and the RNA extracts from clinical NP swabs. The heat-inactivated SARS-CoV-2 control is a commercial product from Twist Bioscience. To prepare saliva with heat-inactivated SARS-CoV-2 control, 4 μL of the control was mixed with 36 μL human saliva, followed by lysate or heat treatment. To prepare saliva with clinical RNA extracts, 30 μL of each RNA extract was mixed with 120 μL human saliva. Then, 28 μL of the saliva sample was used for pH-paper-based RNA extraction, and 140 μL was applied for spin column-based RNA extraction. All the saliva samples could be tested immediately or stored at -80 °C until use.

Colorimetric RT-DAMP Using Cresol Red: The cresol red was dissolved to 50 mM using nuclease-free water. A 2 × non-buffered solution was prepared by combining 20 mM $(NH_4)_2SO_4$, 0.2% (v/v) Tween 20, 100 mM KCl, 8 mM KOH, 2.8 mM dNTP, and 16 mM $MgSO_4$ in a total 500 μL volume. A typical 20-μL colorimetric RT-DAMP reaction using cresol red included 1 × non-buffered solution, 0.2 μM FO, 0.2 μM RO, 1.6 μM FI, 1.6 μM RI, 1.6 μM FC, 1.6 μM RC, 0.3 U μL^{-1} WS RTx, 1.2 U μL^{-1} Bst 2.0 WS, 0.1 mM cresol red, and 1.0 μL target solution. The reactions were incubated at 63 °C for 40 min in Bio-Rad CFX96 Touch Real-Time PCR Detection System. The sequences of the used primers are shown in **Table S1**.

3D-printed Device: The 3D-printed device for pH-paper-based RNA extraction consists of the lid and the container, as shown in **Figure S1**. The lid is designed with a sample inlet in which the top diameter is 12 mm, the bottom diameter is 4 mm, and the height is 35 mm. The container is designed with 48 mm diameter and 4.5 mm thickness. The lid and the container are connected using a screw. Prior to pH-paper-based extraction, the lid is covered by a parafilm with a 4-mm middle hole to tightly fit the inner surface and the sample inlet. The high definition Stereolithographic (SLA) laser-based 3D printer Form 2 from Form labs was used to print the device.

Chemically-heated smart cup and its operation for isothermal amplification: As described in our previous publication,[1] a commercially available Mg–Fe alloy pouch (Innotech Products Ltd., USA) was used to generate chemical heat and phase change material (PCM) (PureTemp 68, Entropy Solutions Inc., Plymouth, MN, USA) was used to regulate the temperature in our smart cup (**Figure S4A**). To incubate the reaction tubes for isothermal amplification, the pouch was, first, placed on a tray into the tray in the 3D printed holder. Subsequently, 7.5 mL of tap water was added into the tray to initiate an exothermic chemical reaction. The generated heat was transferred to the metal tube holder through the PCM. After approximately 10-minute pre-heating, the reaction tubes were inserted into the 3D-printed metal tube holder (**Figure S4B**). After a 40-min incubation, the colorimetric result was immediately read based on the color change of the pH paper disc.

2. Supporting Figures

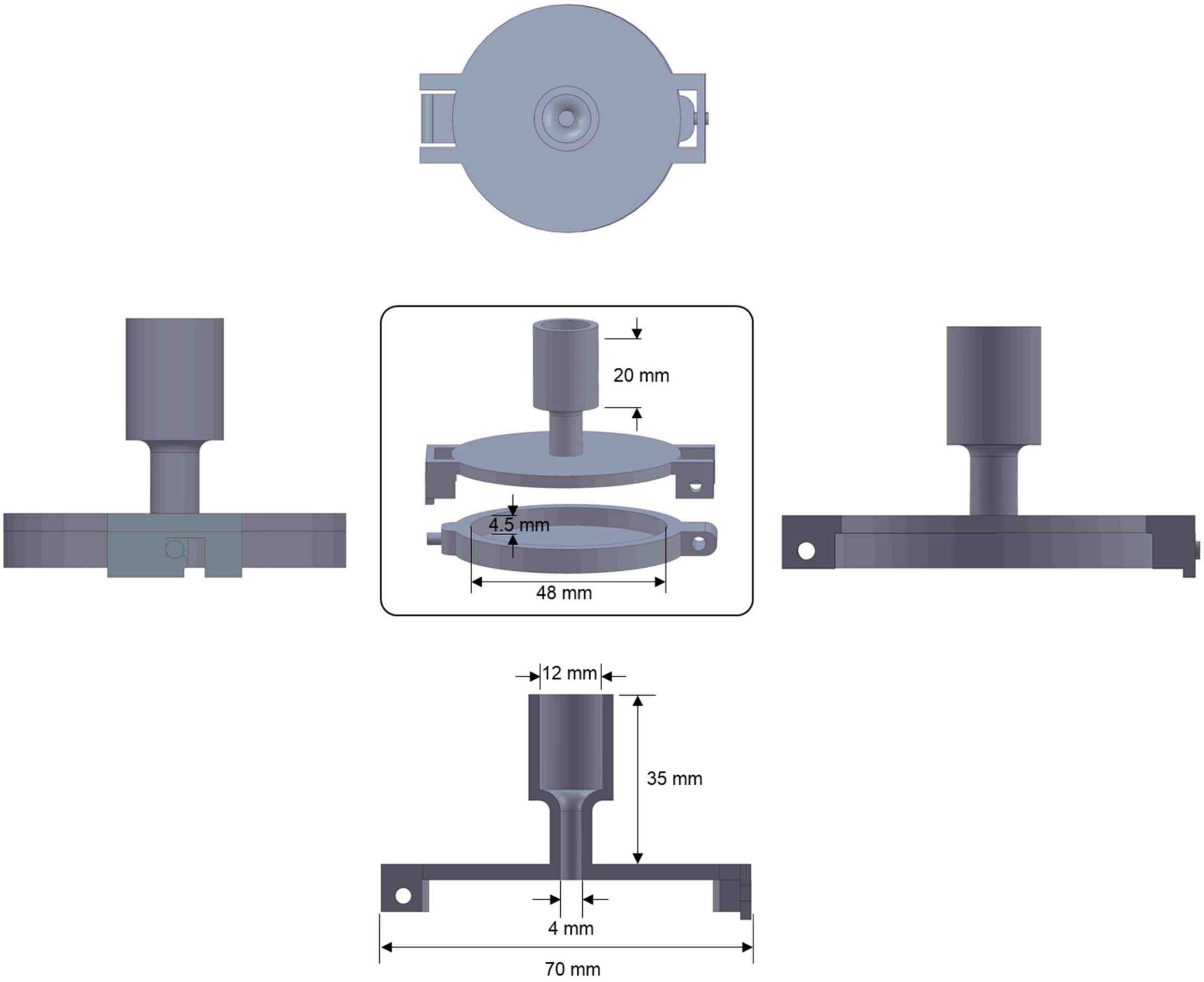

Figure S1. The 3D-printed clamshell device used for pH-paper-based extraction.

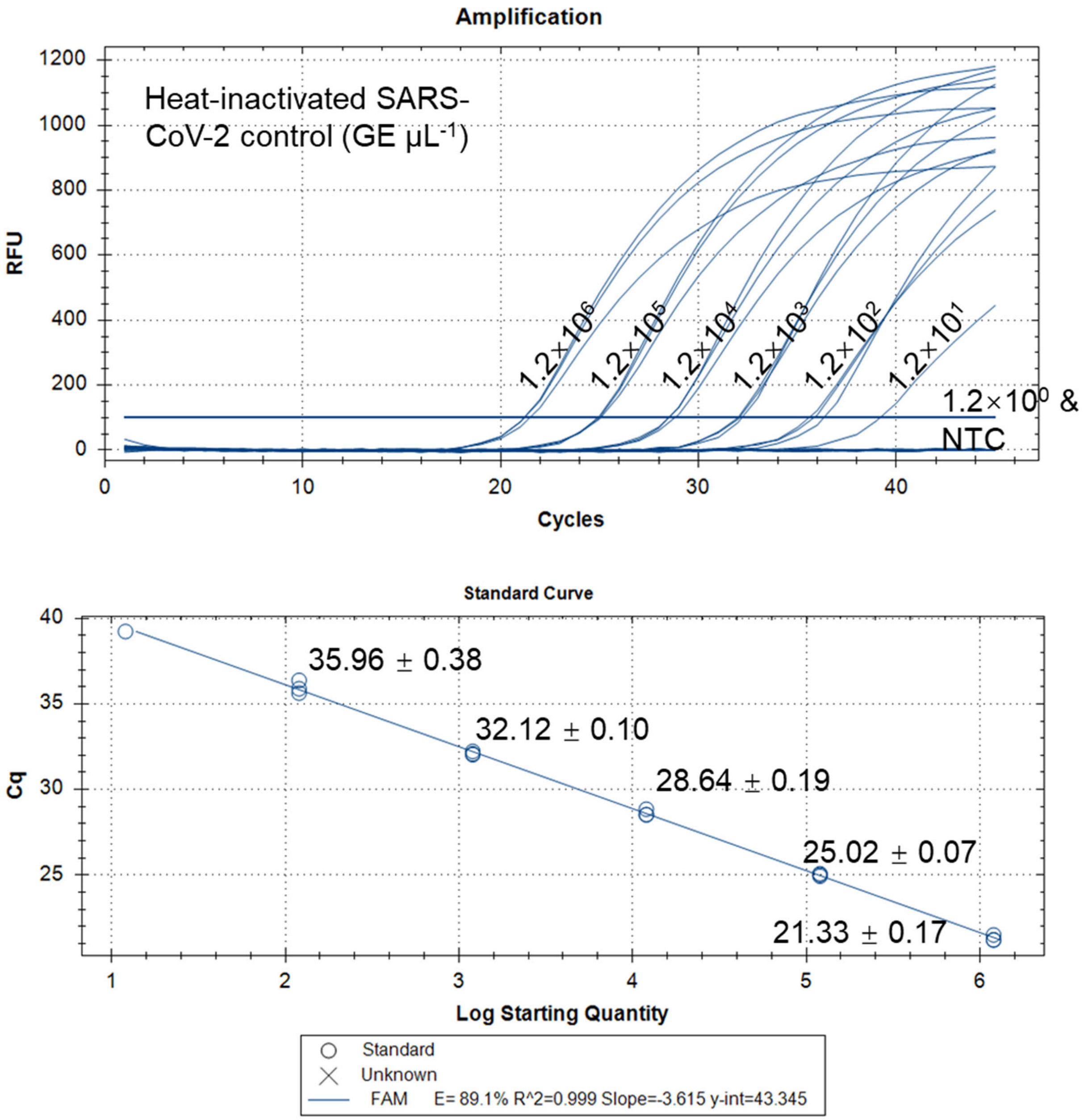

Figure S2. Real-time fluorescence curves of RT-qPCR for testing various concentrations of heat-inactivated SARS-CoV-2 and the linear relationship between Cq and the logarithm with base 10 of target concentrations. GE, genome equivalents. NTC, the solutions without any heat-inactivated SARS-CoV-2. Three replicates were run ($n = 3$). The Cq value indicated was the averaged with the standard deviation of the three replicates.

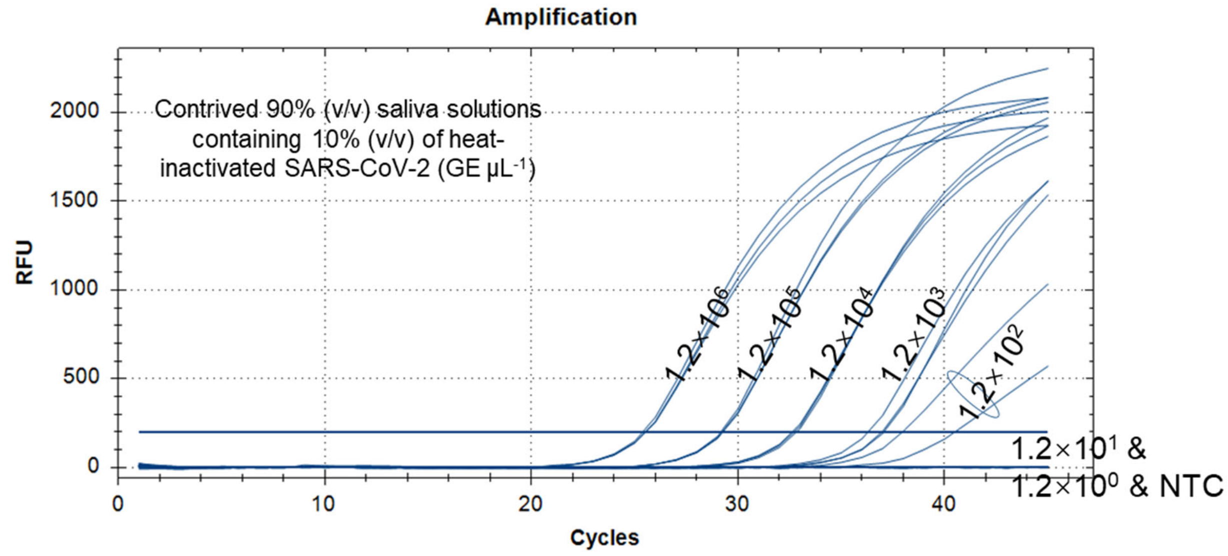

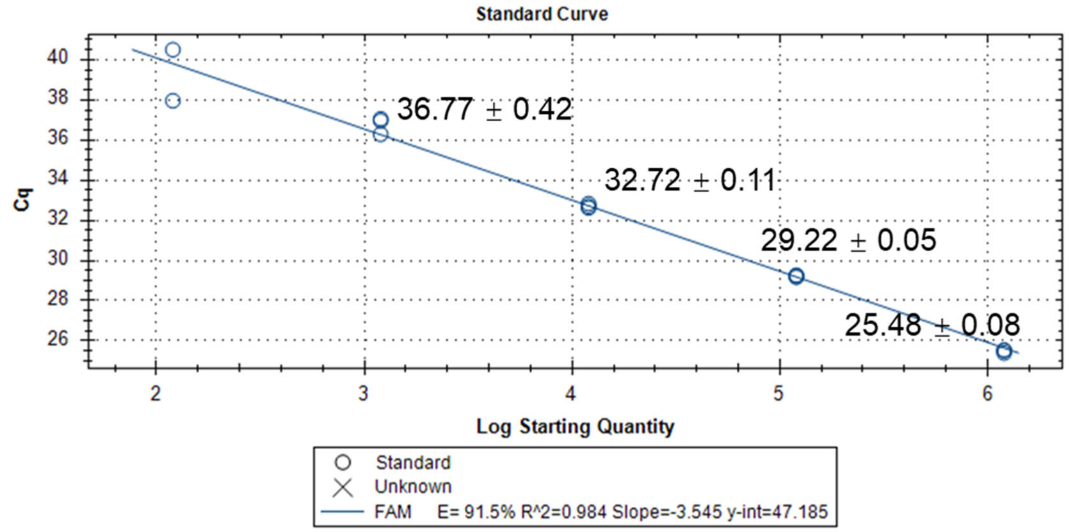

Figure S3. Real-time fluorescence curves of RT-qPCR for testing contrived 90% (v/v) saliva solutions containing 10% (v/v) of heat-inactivated SARS-CoV-2 and the linear relationship between Cq and the logarithm with base 10 of target concentrations. GE, genome equivalents. NTC, the solutions without any heat-inactivated SARS-CoV-2. Three replicates were run (n = 3). The Cq value indicated was the averaged with the standard deviation of the three replicates.

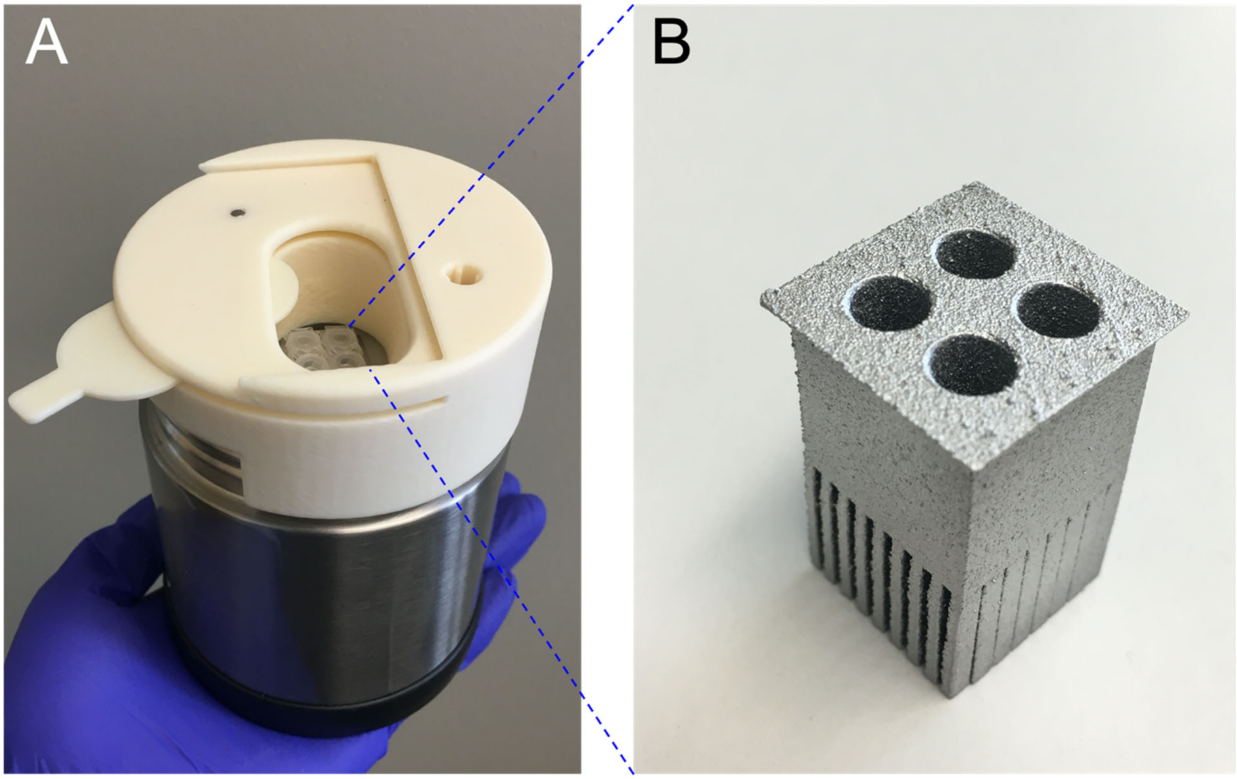

Figure S4. Handheld, chemically-heated, smart cup used for instrument-free SARS-CoV-2 detection. (A) Photograph of the smart cup. (B) Photograph of 3D-printed metal tube holder.

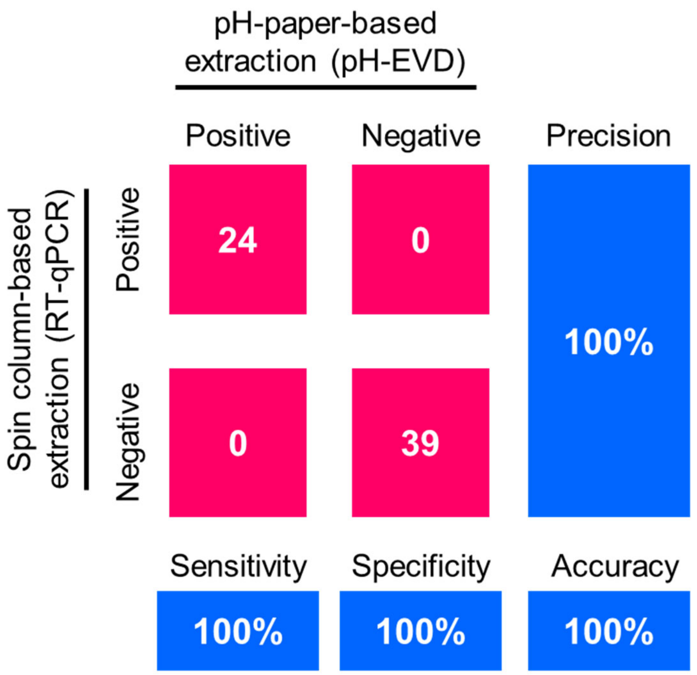

Figure S5. Confusion matrix describing the overall performances of the two assays between positive and negative samples. The cutoff of Cq was 35. The spin column-based extraction and RT-qPCR are considered as the standards.

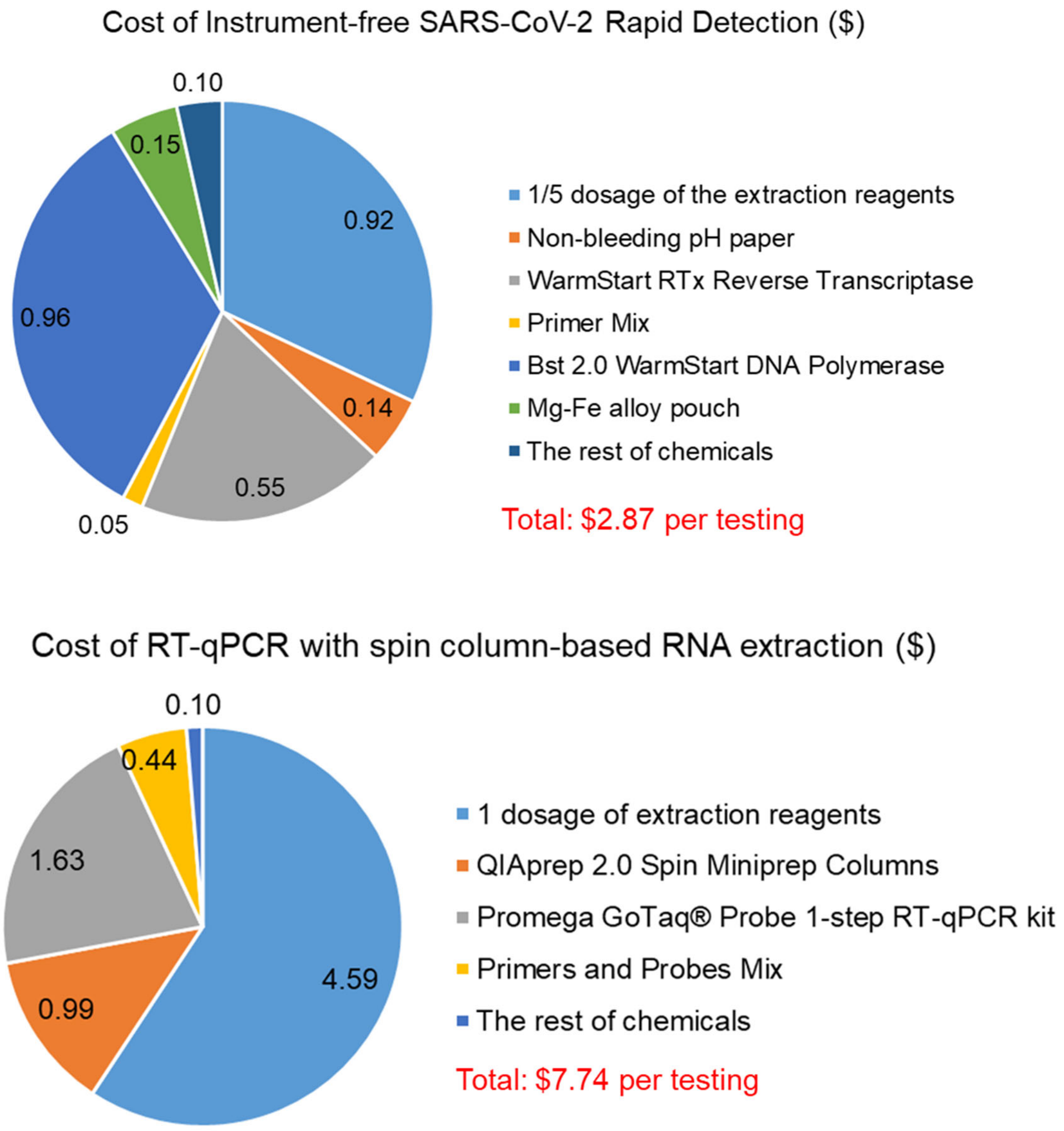

Figure S6. Cost comparison of instrument-free SARS-CoV-2 detection with pH-EVD and the RT-qPCR with spin column-based RNA extraction. The cost only focuses on the commercially available materials and reagents used in this study, not covering equipment fees.

3. Supporting Tables

Table S1. The list of all used primers and probes in this study

Item	Sequence (5’-3’)
RT-DAMP forward outer primer (FO) targeting SARS-CoV-2 N gene	GGCTTCTACGCAGAAGGGA
RT-DAMP reverse outer primer (RO) targeting SARS-CoV-2 N gene	TCTGTCAAGCAGCAGCAAAG
RT-DAMP forward inner primer (FI) targeting SARS-CoV-2 N gene	ACTGTTGCGACTACGTGATGATTTTGTCAAGCCTCTTCTCGTTCC
RT-DAMP reverse inner primer (RI) targeting SARS-CoV-2 N gene	GGAACTTCTCCTGCTAGAATGGCTTTTCAAGAGCAGCATCACCGC
RT-DAMP forward competition primer (FC) targeting SARS-CoV-2 N gene	ACTGTTGCGACTACGTGATGA
RT-DAMP reverse competition primer (RC) targeting SARS-CoV-2 N gene	GGAACTTCTCCTGCTAGAATGGC
nCOV_N1 RT-qPCR forward primer	GACCCCAAAATCAGCGAAAT
nCOV_N1 RT-qPCR reverse primer	TCTGGTTACTGCCAGTTGAATCTG
nCOV_N1 RT-qPCR probe	FAM-ACCCCGCATTACGTTTGGTGGACC-BHQ1

Table S2. Comparison of our pH-paper-based platform and existing paper-based technologies

Target	Paper	Power source for incubation	Visual detection	Limit of detection	Sample-to-answer time	Cost [a]	Ref.
Salmonella typhimurium	FTA card	Battery	Yes	100 CFU/ml in wastewater and egg, and 1000 CFU/ml in milk and juice	About 1 h	Low	[2]
Escherichia coli	FTA card	Battery/ electricity	Yes	10-1000 CFU/mL	About 1 h	Low	[3]
Salmonella typhimurium	Fiber paper-based origami	Battery/ electricity	No	14.2 CFU/mL	About 45 min (only for reaction)	Moderate	[4]
SARS-CoV-2	Chromatography paper	Electricity (qPCR plate)	Yes	200 copies/ reaction	About 80 min	Moderate	[5]
SARS-CoV-2	Chromatography filter paper	Electricity (incubator)	Yes	9.00 ng/μL (0.112 IU/mL) humanized antibody	Less than 30 min	High	[6]
SARS-CoV-2	pH paper	Chemicals (smart cup)	Yes	120 genome equivalents (GE)/μL; 680 copies/ reaction	Less than 46 min	Low	This study

[a] The cost includes that of both reagents and instrument.

References

[1] S. C. Liao, J. Peng, M. G. Mauk, S. Awasthi, J. Song, H. Friedman, H. H. Bau, C. Liu, *Sens Actuators B Chem* **2016**, 229, 232.

[2] R. Tang, H. Yang, Y. Gong, M. You, Z. Liu, J. R. Choi, T. Wen, Z. Qu, Q. Mei, F. Xu, *Lab Chip* **2017**, 17, 1270.

[3] J. R. Choi, J. Hu, R. Tang, Y. Gong, S. Feng, H. Ren, T. Wen, X. Li, W. A. Wan Abas, B. Pingguan-Murphy, F. Xu, *Lab Chip* **2016**, 16, 611.

[4] T. He, J. Li, L. Liu, S. Ge, J. Yu, *RSC Advances* **2020**, 10, 25808.

[5] J. L. Davidson, J. Wang, M. K. Maruthamuthu, A. Dextre, A. Pascual-Garrigos, S. Mohan, S. V. S. Putikam, F. O. I. Osman, D. McChesney, J. Seville, M. S. Verma, *Biosens Bioelectron X* **2021**, 9, 100076.

[6] S. Kasetsirikul, M. Umer, N. Soda, K. R. Sreejith, M. J. A. Shiddiky, N. T. Nguyen, *Analyst* **2020**, 145, 7680.
